# Glial-specific gene alterations associated with manic behaviors

**DOI:** 10.1186/2194-7511-1-20

**Published:** 2013-10-07

**Authors:** Yonglin Gao, Malhar Jhaveri, Zhenmin Lei, Brandy L Chaneb, Jerry Lingrel, Rif S El-Mallakh

**Affiliations:** Mood Disorders Research Program, Department of Psychiatry and Behavioral Sciences, University of Louisville School of Medicine, Louisville, KY 40202 USA; Department of Obstetrics and Gynecology, University of Louisville School of Medicine, Louisville, KY 40202 USA; 645 South Rogers Street, Bloomington, IN 47403 USA; Department of Molecular Genetics, Biochemistry and Microbiology, University of Cincinnati, Cincinnati, OH 45219 USA

**Keywords:** Animal model, Bipolar affective disorder, Glial dysfunction, Lithium, Mania, Sleep deprivation

## Abstract

**Background:**

Glial dysfunction has been purported to be important to the pathophysiology of bipolar illness. However, manic behavior has not been previously demonstrated to result as a consequence of glial pathology. The aim of the current study was to assess the behaviors of the glial-specific sodium pump alpha2 subunit (*ATP1A2*) knockout (KO) heterozygote mice to determine if a glial-specific abnormality can produce manic-like behavior.

**Methods:**

Activity and behavior of hemideficient sodium pump alpha2 KO mice and wild-type (WT) littermates (C57BL6/Black Swiss background) were examined at baseline, following forced swimming stress and restraint stress and after 3 days of sleep deprivation.

**Results and discussion:**

At baseline, the 24-h total distance traveled and center time were significantly greater in KO mice, but there were no behavioral differences with sweet water preference or with inactivity time during forced swim or tail suspension tests. After restraint stress or forced swimming stress, there were no differences in activity. Three days of sleep deprivation utilizing the inverted flowerpot method induced a significant increase in the distance traveled by the KO versus WT mice in the 30-min observation period (p=0.016). Lithium pretreatment has no effect on WT animals versus their baseline but significantly reduces hyperactivity induced by sleep deprivation in KO. Knockout of the glial-specific alpha2 isoform is associated with some manic behaviors compared to WT littermates, suggesting that glial dysfunction could be associated with mania.

## Background

Glial dysfunction has been purported to be important to the pathophysiology of bipolar illness (e.g., Mitterauer 2004, 2011). A multitude of studies have found reduced glial number (Ongür et al. 1998; Rajkowska 2000; Rajkowska et al. 2001; Uranova et al. 2004), reduced glial size (Brauch et al. 2006), and aberrant glial function (Tkachev et al. 2003; Ongür et al. 2008) in bipolar illness compared to non-bipolar controls and involvement of glia in the therapeutic action of effective mood stabilizers (Wang et al. 2012). While indeed there have been demonstrations of glial pathology in bipolar illness, functional or behavioral consequences of that pathology have not been previously demonstrated.

In designing a demonstration of glial dysfunction and consequent behavior, it is important to keep in mind the abnormalities that are known to occur in bipolar patients. It has been previously demonstrated that the alpha2 isoform of the sodium pump (sodium and potassium activated adenosine triphosphatase, or Na,K-ATPase) is reduced in postmortem temporal cortical tissue of bipolar subjects compared to non-bipolar controls (Rose et al. 1998). This is important since the alpha2 subunit is expressed exclusively in glia with the central nervous system (Urayama et al. 1989; Juhaszova and Blaustein 1997a). Consequently, by mimicking the deficiency of the alpha2 subunit in mice, one could simultaneously create a glial abnormality that is known to occur in humans with bipolar disorder.

It has been previously demonstrated that intracerebroventricular (ICV) administration of the specific sodium pump inhibitor, ouabain, may model bipolar disorder (Ruktanonchai et al. 1998; Decker et al. 2000; El-Mallakh et al. 2003). However, this procedure would be expected to inhibit both neuronal alpha3 and glial alpha2 subunits. The preferred approach would be to genetically model the alpha2 deficit observed in the brains of humans with bipolar disorder by examining the behavior in hemideficient alpha2 (*ATP1A2*) knockout mice (Moseley et al. 2007).

## Methods

### Animals

Na,K-ATPase alpha2 knockout (KO) mice were generated at the University of Cincinnati and described by James et al. (1999). KO mice were created upon a Black Swiss background. Homozygous mice die upon birth, but heterozygotes survive and appear grossly normal. Heterozygote mice express about half as much of the alpha2 subunit of the sodium pump as wild-type (WT) littermates. To identify genotypes, PCR genotyping was performed at 4 weeks of age by genomic DNA extracted from the tails. Young adult male and female heterozygote and wild-type littermates were used for the study. All female mice were studied during their estrus period (determined by examination of vaginal smears) (Cooper et al. 1993). Animals were maintained in a 12:12 light/dark cycle and given *ad libitum* food and water. All behavioral testing was done during the light hours of the animals. All experimental procedures were approved by the University of Louisville's Institutional Animal Care and Use Committee (IACUC).

### Experimental procedures

#### Stressors

For the experiments, we investigated motoric activity after a forced swimming stress, a restraint stress, and sleep deprivation. Swim stress was accomplished by placing the mice in 20-cm-deep warm (28°C) water that they are forced to swim continuously for 3 h each day for 3 days in a row. The mice were continuously observed throughout the swim period. Twenty-five animals in each group were studied. Restraint stress was accomplished by placing the animal in a ventilated plastic chamber in which the mouse could not move to any significant degree, for 6 h. Ten animals in each group were studied. Sleep deprivation for 3 days utilized the inverted flowerpot method. Briefly, animals were placed on a small island (3.5 cm diameter) in a pool of water for the entire 72 h period and replaced onto the island whenever they fell into the water. This technique deprives animals of rapid eye movement (REM) sleep (Kitka et al. 2009). Fourteen animals in each group were studied.

The effect of lithium was examined by feeding rodent chow containing 1.994 g lithium/kg of food (Harlan Tekiad, Madison, WI, USA) for 7 days before the stress.

### Experimental procedures

#### Behavior tests

Locomotor activity was performed in a 41.5 cm × 41.5 cm automated, infrared activity monitors (Digiscan, Omnitech Electronics, Columbus, OH, USA). Horizontal activity, total distance, movement time, rest time, vertical activity, margin time, and center time were recorded. The baseline activities were measured for 24 h. Since it is known that sleep deprivation induces a transient motoric hyperactivity previously described as manic-like (Gessa et al. 1995), the activities after previous described stresses were measured for 30 min.

Inactivity time was measured in mice placed in a tank of 25 cm diameter in 20-cm-deep warm water (28°C) for 6 min (Porsolt et al. 1977) and in mice suspended by their tail for 6 min (Steru et al. 1985).

Sweet water preference was carried out by giving the mice the free choice between sodium saccharin-sweetened (Sigma, St. Louis, MO, USA) water (0.1%, 0.5%, or 1%) and tap water supplied in standard drinking bottles in their home cage. The amount of water used over 6-day period was quantified (Hayward et al. 2002).

Serum lithium levels were measured by lithium-sensitive electrode 10 to 12 h after the removal of lithium-containing food.

## Experimental procedures

### Data analysis

A two-tailed *t* test was used to evaluate horizontal movement after forced swimming and restraint stress. ANOVA with a *post hoc* Fisher PLSD was used to analyze all the measures after the sleep deprivation.

## Results

Lithium feeding over a period of 1 week resulted in mean plasma lithium levels of 1.0 mM (range 0.74 to 1.74 mM).

**Figure 1:**
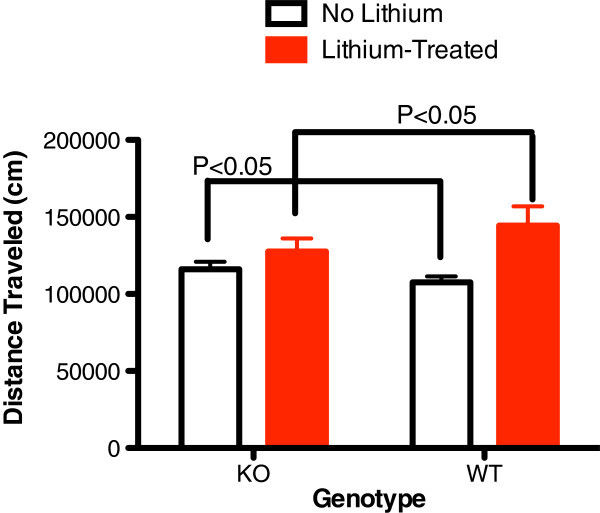
**Baseline 24 h total distance traveled (cm) before and after lithium treatment.** In alpha2 hemideficient KO and WT littermates (*P* < 0.05, *t* = 3.672, *n* = 16). Total distance was significantly greater in KO at baseline and significantly less after lithium treatment relative to WT mice (*P* < 0.05, *t* = 2.901, *n* = 16).

**Figure 2:**
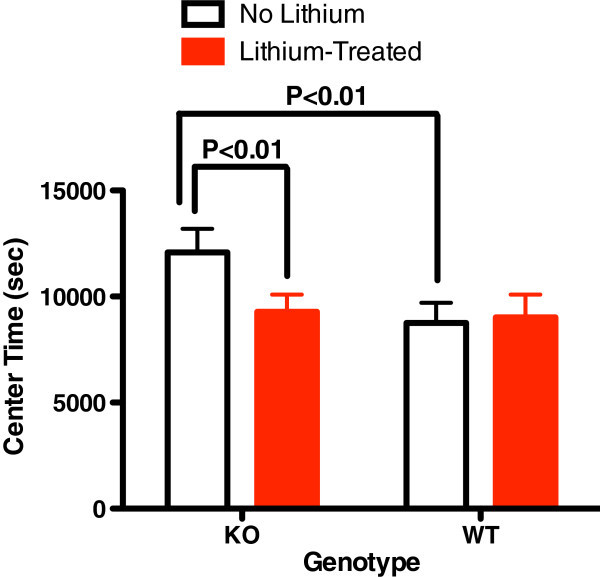
**Baseline 24 h center time (sec) before and after lithium treatment.** In alpha2 hemideficient KO and WT littermates (*P* < 0.01, *t* = 6.288, *n* = 16). Center time was significantly greater in KO at baseline and normalized after lithium treatment relative to WT mice (*P* > 0.05, *t* = 0.574, *n* = 16).

**Figure 3:**
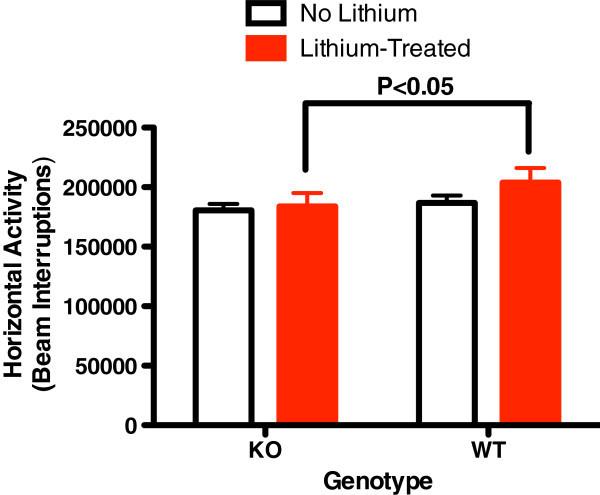
**Baseline 24 h horizontal activity.** The baseline 24-h horizontal activity (number of beam interruptions) was not different between alpha2 hemideficient KO and WT littermates (*P* > 0.05, *t* = 2.121, *n* = 16). However, lithium treatment reduces the activity in KO mice compare to WT mice (*P* < 0.05, *t* = 3.265, *n* = 14).

KO mice had significantly elevated baseline total distance and center time of 24 h in the activity monitor (Figures 1 and 2). Total distance is a measure of exploratory activity. Center time is a measure of risk taking. Horizontal activity, vertical activity, movement time, and rest time were not different. Lithium was associated with normalization of center time in KO animals (Figure 2) and an increase in WT mice but not KO mice (Figures 1 and 2). Horizontal activity was not different at baseline; however, lithium was associated with an increase in horizontal activity in WT mice which did not occur in the KO mice (Figure 3).

After 3 days of 3 h/day of forced swimming, total distance, horizontal activity, and center time were not different in the KO mice compared to their WT littermates. Similarly, 6 h of restraint stress also had no effect on total distance, horizontal activity, and center time.

**Figure 4:**
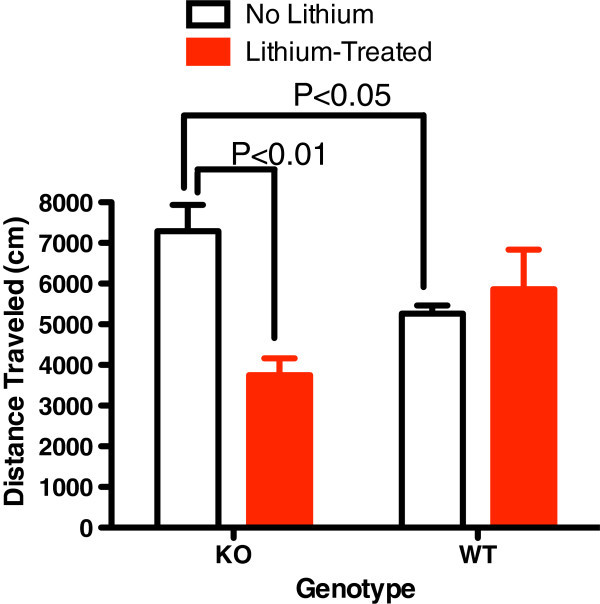
**Sleep deprivation utilizing the inverted flower-pot method results in a significant increase of distance travelled (cm) in 30 min.** By KO mice relative to WT littermates (*P* < 0.05, *n* = 5 to 7 mice/group). This was significantly reduced with lithium treatment in KO mice only.

**Figure 5:**
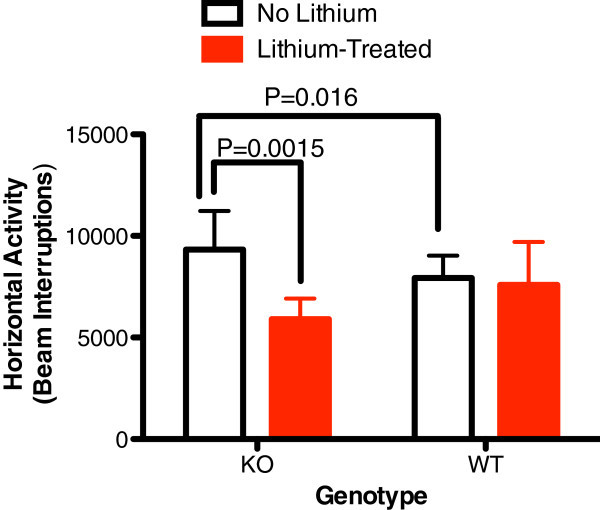
**Sleep deprivation utilizing the inverted flower-pot method results in a significant increase of Horizontal activity over 30 min.** In KO mice relative to WT littermates (*n* = 5 to 7 mice/group). This was significantly reduced with lithium treatment in KO mice only.

After 72 h of sleep deprivation utilizing the inverted flowerpot method, total distance traveled and horizontal activity were significantly increased in KO mice (Figures 4 and 5). Lithium lowered postsleep deprivation activity only in KO mice (Figures 4 and 5).

Other measures such as sweet water preference, inactivity time with forced swim test, and tail suspension test were not different at baseline between the KO and WT.

## Discussion

This is the first demonstration that abnormalities associated with glial dysfunction can produce behavioral abnormalities consistent with mania. Na,K-ATPase alpha2 KO mice exhibited some increased exploratory activity (total distance travelled, Figure 1) and risk taking behavior (center time, Figure 2) at baseline. Additionally, REM sleep deprivation of KO mice was associated with an increase in walking activity (Figure 4) and horizontal activity (Figure 5), resembling the increase in goal-directed activity that occurs with mania after sleep deprivation in humans with bipolar illness. All of the observed differences in the KO mice were normalized with lithium treatment, i.e., after lithium treatment, KO mice resembled the untreated WT animals (Figures 1, 2, 3, 4, and 5), as frequently occurs in humans with bipolar disorder. These changes suggest that hemi-expression of the alpha2 subunit of the Na,K-ATPase in mice (James et al. 1999) - similar to what occurs in the brain of human bipolar (Rose et al. 1998) - produces behavior in mice that is compatible with mania.

In rodents, the alpha2 isoform accounts for about 20% of all alpha subunit expression while the ubiquitous alpha1 subunit accounts for the remaining 80% (Golovina et al. 2003a). The hemideficient KO mice express approximately half as many pump units in the membrane as WT littermates (Golovina et al. 2003a), which is very similar to the findings in the postmortem temporal cortex of bipolar disorder patients (Rose et al. 1998). The function of these two isoforms appears distinct, so that in the mice and in bipolar patients, there is no upregulation of the alpha1 expression to compensate for the deficiency in alpha2 expression (Rose et al. 1998; Golovina et al. 2003a). Glia, in general, and the alpha2 subunit in particular are important in modulating the synaptic environment and calcium signaling in the synapse (Golovina et al. 2003a; Xiong and Stringer 2000). Additionally, the alpha2 subunit is not uniformly distributed throughout the plasma membrane of astrocytes; rather, it is intimately congregated in microdomains that overlie calcium-rich endoplasmic reticulum (Juhaszova and Blaustein 1997a, b). Consequently, alterations in the alpha2 isoform expression alter sodium and calcium flux (Golovina et al. 2003a, b). Furthermore, the metabolic activity of glia and neurons are coupled; it is the glycolytic activity of the glial Na,K-ATPase that produces the lactate that fuels the neuronal energy demands associated with firing and neurotransmission (Magistretti 2006, 2009) particularly at times of neuronal distress (Schurr et al. 1997; Schurr and Rigor 1998). The current study suggests that glial dysfunction induced by underexpression of the alpha2 subunit of the Na,K-ATPase, in both the KO mice and humans with bipolar illness (Rose et al. 1998), may be associated with some manic symptoms.

While hyperactivity was observed at baseline in the KO mice, it is notable that sleep deprivation also induced hyperactive behavior in the KO mice compared to WT littermates. Abnormalities of sleep are common and important in the pathophysiology of bipolar illness (Brill et al. 2011; Plante and Winkelman 2008), and sleep and circadian rhythm-related genes appear to be associated with bipolar disorder (Mansour et al. 2009; Sjoholm et al. 2010). Sleep deprivation can trigger mood episode switches in patients with bipolar disorder (Salvadore et al. 2010). Bipolar patients appear to be sensitive to cycle or rhythm disruption (Goodwin and Jamison 1990). Sleep deprivation is believed to play a role in the induction of mania (Plante and Winkelman 2008) but is used also as a treatment for bipolar depression (Wu et al. 2009). There are different types of sleep deprivation, and in bipolar disorder, REM sleep deprivation appears to be closely related to induction of mood elevation (Albert et al. 1970; Vogel et al. 1975; Salvadore et al. 2010). REM density is increased in euthymic bipolar subjects compared to normal controls (Talbot et al. 2009) suggesting a greater need in bipolar patients. It is notable that sleep deprivation by inverted flowerpot method, which prevents REM stage sleep (Kitka et al. 2009), was the only stressor that induced lithium-preventable hyperactivity in the KO mice (Figures 4 and 5).

There are clear limitations to the current study. The primary one is that we did not directly document that the glia within the KO mice are dysfunctional. However, multiple *ex vivo* examinations of the same KO mouse model reveals that glial function is compromised in a significant manner (Golovina et al. 2003a, b; Hartford et al. 2004). It is reasonable to assume that the differences in behavior observed are either directly or indirectly related to the only difference between the KO mice and their WT littermates. Additionally, it should be noted that the KO mice were created on a Black Swiss strain background. Black Swiss mice have baseline higher level of activity than other strains and were felt to be ‘a good choice for modeling several domains of mania’ by Flaisher-Grinberg and Einat (2010), who investigated strain-specific mouse behavior. But, all of our experiments were designed to examine KO mice compared to their wild-type littermates. Consequently, the increases in activity seen in KOs and the relative reduction in hyperactivity induced in KOs compared to wild-type littermates exhibit hyperactivity and manic-like behavior that is above and beyond that observed by Flaisher-Grinberg and Einat (2010). Finally, the behavioral changes observed are not consistent with the full syndrome of mania; only locomotor activity showed change in alpha2 KO mice. Bipolar mania is manifested by several symptoms, such as irritability, reduced need for sleep, or increased distractibility, which can be measured in animals. This would suggest that the specific aspect of glial dysfunction modeled herein (reduction of Na,K-ATPase alpha2 expression) does not produce the full syndrome of mania but is associated with some aspects of the behavior.

## Conclusions

In summary, this is the first demonstration that targeted glial anomaly, in a manner that mimics findings in people with bipolar disorder, can produce behavioral changes in rodents consistent with ‘mania’. This is an important step in understanding the potential role of a wide range of glial abnormalities in bipolar illness. Future investigation of glial abnormalities would appear to be an important goal to understand the pathophysiology of bipolar illness.

## Abbreviations

ANOVA: Analysis of variance
ATP1A2: The gene name for the alpha2 subunit of the Na,K-ATPase
DNA: Deoxyribonucleic acid
IACUC: Institutional animal care and use committee
KO: Knockout
Na: K-ATPase: The sodium- and potassium-activated adenosine triphosphatase (enzyme pump)
PCR: Polymerase chain reaction
PLSD: Protected least significant difference
REM: Rapid eye movement (sleep)
WT: Wild type.

## References

[CR1] Albert I, Cicala GA, Siegel J (1970). The behavioral effects of REM sleep deprivation in rats. Psychophysiol.

[CR2] Brauch RA, Adnan El-Masri M, Parker JC, El-Mallakh RS (2006). Glial cell number and neuron/glial cell ratios in postmortem brains of bipolar individuals. J Affect Dis.

[CR3] Brill S, Penagaluri P, Roberts RJ, Gao Y, El-Mallakh RS (2011). Sleep disturbances in euthymic bipolar patients. Ann Clin Psychia.

[CR4] Cooper RL, Goldman JM, Vandenbergh JG, Chapin RE, Heindel J (1993). Monitoring the estrous cycle in the laboratory rodent by vaginal lavage. Methods in Toxicology: Male Reproductive Toxicology.

[CR5] Decker S, Grider G, Cobb M, Li XP, Huff MO, El-Mallakh RS, Levy RS (2000). Open field is more sensitive than automated activity monitor in documenting ouabain-induced hyperlocomotion in the development of an animal model for bipolar illness. Prog Neuropsycho Biol Psychia.

[CR6] El-Mallakh RS, El-Masri MA, Huff MO, Li XP, Decker S, Levy RS (2003). Intracerebroventricular administration of ouabain as a model of mania in rats. Bipo Dis.

[CR7] Flaisher-Grinberg S, Einat H (2010). Strain-specific battery of tests for domains of mania: effects of valproate, lithium and imipramine. Front Psychia.

[CR8] Gessa GL, Pani L, Fadda P, Fratta W (1995). Sleep deprivation in the rat: an animal model of mania. Eur Neuropsycho.

[CR9] Golovina VA, Song H, James PF, Lingrel JB, Blaustein MP (2003). Na+ pump α2-subunit expression modulates Ca2+ signaling. Am J Physiol Cell Physiol.

[CR10] Golovina V, Song H, James P, Lingrel J, Blaustein M (2003). Regulation of Ca2+ signaling by Na+ pump alpha-2 subunit expression. Ann N Y Acad Sci.

[CR11] Goodwin F, Jamison K (1990). Manic-Depressive Illness.

[CR12] Hartford AK, Messer ML, Moseley AE, Lingrel JB, Delamere NA (2004). Na, K-ATPase alpha 2 inhibition alters calcium responses in optic nerve astrocytes. Glia.

[CR13] Hayward MD, Pintar JE, Low MJ (2002). Selective reward deficit in mice lacking beta-endorphin and enkephalin. J Neurosci.

[CR14] James PF, Grupp IL, Grupp G, Woo AL, Askew GR, Croyle ML, Walsh RA, Lingrel JB (1999). Identification of a specific role for the Na, K-ATPase alpha 2 isoform as a regulator of calcium in the heart. Mol Cell.

[CR15] Juhaszova M, Blaustein MP (1997). Na+ pump low and high ouabain affinity alpha subunit isoforms are differently distributed in cells. Proc Natl Acad Sci USA.

[CR16] Juhaszova M, Blaustein MP (1997). Distinct distribution of different Na+ pump alpha subunit isoforms in plasmalemma. Physiological implications. Ann NY. Acad Sci.

[CR17] Kitka T, Katai Z, Pap D, Molnar E, Adori C, Bagdy G (2009). Small platform sleep deprivation selectively increases the average duration of rapid eye movement sleep episodes during sleep rebound. Behav Brain Res.

[CR18] Magistretti PJ (2006). Neuron-glia metabolic coupling and plasticity. J Exp Biol.

[CR19] Magistretti PJ (2009). Role of glutamate in neuron-glia metabolic coupling. Am J Clin Nutr.

[CR20] Mansour HA, Talkowski ME, Wood J, Chowdari KV, McClain L, Prasad K, Montrose D, Fagiolini A, Friedman ES, Allen MH, Bowden CL, Calabrese J, El-Mallakh RS, Escamilla M, Faraone SV, Fossey MD, Gyulai L, Loftis JM, Hauser P, Ketter TA, Marangell LB, Miklowitz DJ, Nierenberg AA, Patel J, Sachs GS, Sklar P, Smoller JW, Laird N, Keshavan M, Thase ME (2009). Association study of 21 circadian genes with bipolar I disorder, schizoaffective disorder, and schizophrenia. Bipolar Dis.

[CR21] Mitterauer BJ (2004). Imbalance of glial-neuronal interaction in synapses: a possible mechanism of the pathophysiology of bipolar disorder. Neurosci.

[CR22] Mitterauer BJ (2011). Downregulation and upregulation of glial connexins may cause synaptic imbalances responsible for the pathophysiology of bipolar disorder. CNS Neurosci Ther.

[CR23] Moseley AE, Williams MT, Schaefer TL, Bohanan CS, Neumann JC, Behbehani MM, Vorhees CV, Lingrel JB (2007). Deficiency in Na, K-ATPase alpha isoform genes alters spatial learning, motor activity, and anxiety in mice. J Neurosci.

[CR24] Ongür P, Drevets WC, Price JL (1998). Glial reduction in the subgenual prefrontal cortex in mood disorders. Proc Natl Acad Sci USA.

[CR25] Ongür D, Jensen JE, Prescot AP, Stork C, Lundy M, Cohen BM, Renshaw PF (2008). Abnormal glutamatergic neurotransmission and neuronal-glial interactions in acute mania. Biol Psychia.

[CR26] Plante DT, Winkelman JW (2008). Sleep disturbance in bipolar disorder: therapeutic implications. Am J Psychia.

[CR27] Porsolt RD, Bertin A, Jalfre M (1977). Behavioral despair in mice: a primary screening test for antidepressants. Arch Int Pharmacodyn Ther.

[CR28] Rajkowska G (2000). Postmortem studies in mood disorders indicate altered numbers of neurons and glial cells. Biol Psychia.

[CR29] Rajkowska G, Halaris A, Selemon LD (2001). Reductions in neuronal and glial density characterize the dosolateral prefrontal cortex in bipolar disorder. Biol Psychia.

[CR30] Rose AM, Mellett BJ, Valdes R, Kleinman JE, Herman MM, Li R, El-Mallakh RS (1998). Alpha 2 isoform of the Na, K-adenosine triphosphatase is reduced in temporal cortex of bipolar individuals. Biol Psychia.

[CR31] Ruktanonchai DJ, El-Mallakh RS, Li R, Levy RS (1998). Persistent hyperactivity following a single intracerebroventricular dose of ouabain. Physiol Behav.

[CR32] Salvadore G, Quiroz JA, Machado-Vieira R, Henter ID, Manji HK, Zarate CA (2010). The neurobiology of the switch process in bipolar disorder: a review. J Clin Psychia.

[CR33] Schurr A, Rigor BM (1998). Brain anaerobic lactate production: a suicide note or a survival kit?. Dev Neurosci.

[CR34] Schurr A, Payne RS, Miller JJ, Rigor BM (1997). Glia are the main source of lactate utilized by neurons for recovery of function posthypoxia. Brain Res.

[CR35] Sjoholm LK, Backlund L, Cheteh EH, Ek IR, Frisen L, Schalling M, Osby U, Lavebratt C, Nikamo P (2010). CRY2 is associated with rapid cycling in bipolar disorder patients. PLoS One.

[CR36] Steru L, Chermat R, Thierry B, Simon P (1985). The tail suspension test: a new method for screening antidepressants in mice. Psychopharmacology (Berl).

[CR37] Talbot LS, Hairston IS, Eidelman P, Gruber J, Harvey AG (2009). The effect of mood on sleep onset latency and REM sleep in interepisode bipolar disorder. J Abnorm Psychol.

[CR38] Tkachev D, Mimmack ML, Ryan MM, Wayland M, Freeman T, Jones PB, Starkey M, Webster MJ, Yolken RH, Bahn S (2003). Oligodendrocyte dysfunction in schizophrenia and bipolar disorder. Lancet.

[CR39] Uranova NA, Vostrikov VM, Orlovskaya DD, Rachmanova VI (2004). Oligodendroglial density in the prefrontal cortex in schizophrenia and mood disorders: a study from the Stanley neuropathology consortium. Schizophr Res.

[CR40] Urayama O, Shutt H, Sweadner KJ (1989). Identification of three isozyme proteins of the catalytic subunit of the Na, K-ATPase in rat brain. J Biol Chem.

[CR41] Vogel GW, Thurmond A, Gibbons P, Sloan K, Walker M (1975). REM sleep reduction effects on depression syndromes. Arch Gen Psychia.

[CR42] Wang CC, Chen PS, Hsu CW, Wu SJ, Lin CT, Gean PW (2012). Valproic acid mediates the synaptic excitatory/inhibitory balance through astrocytes–a preliminary study. Prog Neuropsycho Biol Psychia.

[CR43] Wu JC, Kelsoe JR, Schachat C, Bunney BG, DeModena A, Golshan S, Gillin JC, Potkin SG, Bunney WE (2009). Rapid and sustained antidepressant response with sleep deprivation and chronotherapy in bipolar disorder. Biol Psychia.

[CR44] Xiong Z-Q, Stringer JL (2000). Sodium pump activity, Not glial spatial buffering, clears potassium after epileptiform activity induced in the dentate gyrus. J Neurophysiol.

